# Boosting Drug Discovery for Parkinson’s: Enhancement of the Delivery of a Monoamine Oxidase-B Inhibitor by Brain-Targeted PEGylated Polycaprolactone-Based Nanoparticles

**DOI:** 10.3390/pharmaceutics11070331

**Published:** 2019-07-12

**Authors:** Miguel Pinto, Carlos Fernandes, Eva Martins, Renata Silva, Sofia Benfeito, Fernando Cagide, Ricardo F. Mendes, Filipe A. Almeida Paz, Jorge Garrido, Fernando Remião, Fernanda Borges

**Affiliations:** 1 CIQUP, Departmento de Química e Bioquímica, Centro de Investigação em Química, Faculdade de Ciências, Universidade do Porto, 4169-007 Porto, Portugal; 2 UCIBIO-REQUIMTE, Laboratório de Toxicologia, Departamento de Ciências Biológicas, Faculdade de Farmácia, Universidade do Porto, 4050-313 Porto, Portugal; 3 Departamento de Química, CICECO-Instituto de Materiais de Aveiro, Universidade de Aveiro, 3810-193 Aveiro, Portugal; 4 Departamento de Engenharia Química, Instituto Superior de Engenharia do Porto (ISEP), Instituto Politécnico do Porto, 4200-072 Porto, Portugal

**Keywords:** Parkinson disease, chromone, monoamine oxidase B inhibitor, PEGylated nanoparticles, intestinal and brain permeability

## Abstract

The current pharmacological treatments for Parkinson’s disease only offer symptomatic relief to the patients and are based on the administration of levodopa and catechol-O-methyltransferase or monoamine oxidase-B inhibitors (IMAO-B). Since the majority of drug candidates fail in pre- and clinical trials, due largely to bioavailability pitfalls, the use of polymeric nanoparticles (NPs) as drug delivery systems has been reported as an interesting tool to increase the stealth capacity of drugs or help drug candidates to surpass biological barriers, among other benefits. Thus, a novel potent, selective, and reversible IMAO-B (chromone C27, IC_50_ = 670 ± 130 *p*M) was encapsulated in poly(caprolactone) (PCL) NPs by a nanoprecipitation process. The resulting C27-loaded PEGylated PCL NPs (~213 nm) showed high stability and no cytotoxic effects in neuronal (SH-SY5Y), epithelial (Caco-2), and endothelial (*h*CMEC/D3) cells. An accumulation of PEGylated PCL NPs in the cytoplasm of SH-SY5Y and *h*CMEC/D3 cells was also observed, and their permeation across Caco-2 and *h*CMEC/D3 cell monolayers, used as in vitro models of the human intestine and blood-brain barrier, respectively, was demonstrated. PEGylated PCL NPs delivered C27 at concentrations higher than the MAO-B IC_50_ value, which provides evidence of their relevance to solving the drug discovery pitfalls.

## 1. Introduction

Parkinson’s disease (PD) is a multifactorial disorder characterized by the progressive degeneration of the structure and well-function of the central nervous system (CNS), leading to a depletion of dopaminergic neurons in the substantia nigra [1]. The decrease of the neurotransmitter dopamine in neuron cleft is associated with movement control and cognitive losses, which appear as a form of tremors, memory loss, and inconsistent speech. Nowadays, PD is the second most prevalent neurodegenerative disorder, affecting 1–2% of the world’s population above 65 years of age, increasing to approximately 4% in individuals above 85 years of age [2].

Despite how clinical trials for PD have long relied on observing whether a therapy improves the symptomatology of patients, the studies performed so far were not able to reveal information about how the treatment affects the progressive neurodegeneration process. The current pharmacological treatments only offer symptomatic relief to the patients [3], and are based on the administration of levodopa and catechol-O-methyltransferase or monoamine oxidase B inhibitors (IMAO-B) [4]. 

Monoamine oxidase-B (MAO-B) is one of the isoforms of monoamine oxidases involved in the metabolization of dopamine in neuronal tissues, whose expression increases about 4-fold with aging. Therefore, IMAO-Bs are used to decrease the turnover rate of striatal dopamine in early PD, or as an adjunctive therapy in patients treated with levodopa that are experiencing motor complications [3]. However, until now, no IMAO-B developed so far has been able to modify or revert the progression of PD [5]. 

**Figure 1:**
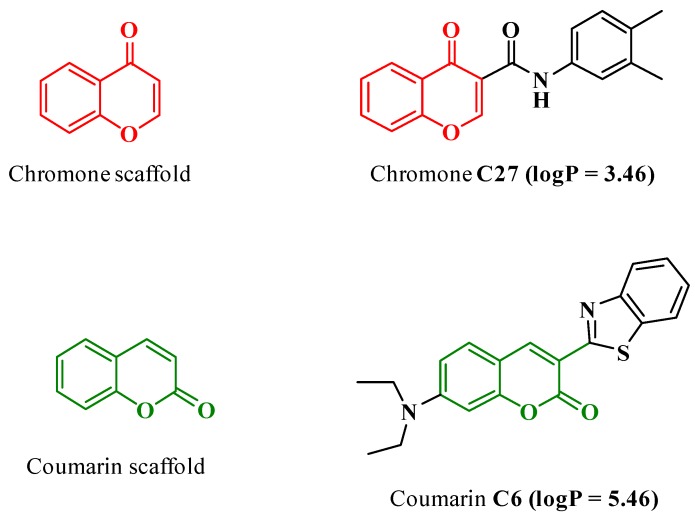
Chemical structures of chromone C27 and coumarin C6. The values of logP were obtained from the literature [5,9].

As a result, in recent years, an intensive search focused on the discovery of novel IMAO-B has been carried out and, in line, chromone has emerged as a validated scaffold (Figure 1) for the development of novel MAO inhibitors [6,7]. In fact, a chromone-based compound (Figure 1, C27) has been reported as a potent, selective, and reversible IMAO-B (IC_50_ = 670 ± 130 *p*M) [5]. This IC_50_ value is one order of magnitude lower than the reference inhibitors ((R)-(−)-deprenyl (IC_50_ = 16.73 ± 1.48 nM), rasagiline (IC_50_ = 49.66 ± 2.26 nM) and safinamide (IC_50_ = 23.07 ± 2.07 nM) [8]. Despite C27’s remarkable outline, several setbacks, mainly related to poor water solubility and bioavailability, have hampered the progress toward in vivo preclinical studies. 

To overcome the attrition rates of central nervous system (CNS) drug discovery and development programs, new precision nanomedicine approaches are being developed. The main goals of these new approaches are the increment of stability, solubility, and other tunable properties and therapeutic index, namely, by improving the targeted delivery across the blood-brain barrier (BBB), of neuroactive drugs and drug candidates. 

Through the years, nanoparticles (NPs) based on biodegradable polymers have been used for controlled drug delivery and to improve the therapeutic performance of drugs. Recently, they have been also used to solve shortcomings related to the solubility, stability, and bioavailability of drug candidates and other bioactive molecules [10,11]. Among the various biodegradable polymers approved by the US Food and Drug Administration, poly(lactide) (PLA), poly(d,l-lactide-*co*-glycolide) (PLGA), and poly(caprolactone) (PCL) are the most reported in the literature [12]. In particular, PCL is described as a non-toxic semi-crystalline hydrophobic polyester, miscible with a variety of polymers, with a high toughness and biocompatibility, since, in physiological conditions, it degrades slower than other biodegradable polyesters [13]. The surface of NPs can be coated with PEGylated surfactants, such as polysorbate 80 (Tween^®^ 80, T80) or poloxamers [8,14], to increase the permeability of NPs into the brain [15]. This process is described to be related to an improvement of the blood circulation time and to the stealth nature of PEG-coated NPs [16]. The PEGylated moiety shields the surface from aggregation, opsonization, and phagocytosis, ensuring the NPs remain undetected by the reticuloendothelial system and increasing their blood circulation half-life [17]. In line with this, Wilson et al. used NPs coated with T80 to deliver rivastigmine and tacrine in rat brains [18,19]. PCL-T80 NPs were also reported to be internalized into glioma C6 cells, with a higher cellular uptake than the PCL NPs, and were able to successfully deliver a nerve growth factor into outbred C57BL/6 mice brain [20,21]. Furthermore, Wang et al. proved that PCL-T80 NPs were capable of delivering paclitaxel in the brains of male Sprague–Dawley rats after intravenous injection [22].

A handful of studies have addressed the use of PEGylated NPs to tackle drug discovery and development problems in the field of neurodegenerative diseases [23]. Even though rasagiline and selegiline (IMAO-B used in therapy) were effectively encapsulated in polymeric NPs [3,24], a gap still exists on the use of nanotechnology to solve site-targeted and absorption, distribution, metabolism, excretion, and toxicity (ADMET) problems along the pre-clinical phase. Herein, the IMAO-B chromone C27 was encapsulated into PCL NPs coated with T80 and a nanoformulation with suitable morphological and physicochemical properties was obtained after an optimization process. The release profile of C27 from PEGylated PCL NPs was evaluated, as well as the unloaded and loaded NPs cytotoxicity outline in human differentiated neuroblastoma (SH-SY5Y), epithelial colorectal adenocarcinoma (Caco-2), and endothelial brain (*h*CMEC/D3) cells. Moreover, cellular uptake (in SH-SY5Y and *h*CMEC/D3 cells) and permeability studies (in Caco-2 and *h*CMEC/D3 cells) were performed. For the cellular uptake, intracellular localization, and permeability studies, PEGylated PCL NPs containing a fluorescent coumarin-based probe (coumarin-6 dye, Figure 1) were also prepared. 

## 2. Materials and Methods 

### 2.1. Materials and Reagents

Polycaprolactone (PCL, *M_n_* ≈ 10,000 Da, determined by gel permeation chromatography) and polysorbate 80 (T80) were purchased from Sigma-Aldrich (Sintra, Portugal) and used without further purification. The reagents and solutions used for cell-based assays were described in [8] and are detailed in the Supplementary Materials. EndoGRO Media was acquired from Merck (Cambridge, MA, USA) and rat tail collagen type I (low viscosity) was purchased from Cultrex. Other reagents were obtained from Sigma-Aldrich (Sintra, Portugal). The water used was Milli-Q filtered (Millipore, Burlington, MA, USA).

### 2.2. Synthesis of N-(3′,4′-Dimethylphenyl)-4-oxo-4H-chromene-3-carboxamide (C27)

The synthesis of *N*-(3′,4′-dimethylphenyl)-4-oxo-4*H*-chromene-3-carboxamide (C27) was previously reported [5]. The structural characterization and purity were ascertained by nuclear magnetic resonance (^1^H NMR, ^13^C NMR and DEPT) (data available in the Supplementary Materials).

### 2.3. Preparation of PEGylated PCL-Based NPs

The encapsulation of chromone C27 in PEGylated PCL-based NPs was performed using the nanoprecipitation method [8]. The nanoformulation was prepared by dissolving PCL and C27 (2.5, 5 or 10% of PCL weight) in acetone and adding it dropwise to Milli-Q water containing 0.2% T80 under vigorous magnetic stirring. The resulting suspension was left under stirring for 1 h at room temperature. Acetone was then fully eliminated by evaporation under reduced pressure at room temperature. C27 loaded NPs were purified by ultrafiltration (10 min, 3000× *g*, Amicon Ultra 100 kDa MWCO, Millipore) and stored at 4 °C until use. The unloaded NPs were similarly prepared, as described above.

The encapsulation of coumarin-6 (C6) in PCL NPs was performed in a similar manner to that described above (1% *w*/*w* of PCL).

For simplicity, from this point onwards PCL nanoformulations containing C27 and C6 will be labelled PCL@C27 NPs and PCL@C6 NPs, respectively. Unloaded NPs will be referred to as PCL NPs. 

### 2.4. Encapsulation and Drug Loading Efficiency

The quantification of chromone C27 was performed using a Shimadzu UV-Vis spectrophotometer (UV-1700 PharmaSpec, Kyoto, Japan). The C27 UV/Vis spectra were obtained using a C27 solution (50 µM) prepared in dimethyl sulfoxide (DMSO). The amount of C27 incorporated into the PCL@C27 NPs was determined directly after the complete dissolution of NPs in DMSO. The encapsulation efficiency (EE%) was calculated as the ratio between the chromone content in the freeze-dried powder and the initial chromone amount used in the NPs preparation (Equation (1)) [25]. The drug loading capacity (DLC%) was determined as the ratio between the amount of C27 encapsulated and the mass of NP powder (Equation (2)) [26].

(1) $$\mathrm{EE}\;\%=\frac{\mathrm{Amount}\;\mathrm{of}\;\mathrm{loaded}\;C27}{\mathrm{Amount}\;\mathrm{of}\;\mathrm{feeding}\;C27}*100\%;$$

(2) $$\mathrm{DLC}\;\%=\frac{\mathrm{Amount}\;\mathrm{of}\;\mathrm{loaded}\;C27}{\mathrm{Amount}\;\mathrm{of}\;\mathrm{PCL}@C27\;\mathrm{NPs}}*100\%.$$

Briefly, 1 mL of nanosuspension was centrifuged (15 min, 16,060× *g*, 4 °C) and washed three times with Milli-Q water. Then, the pellet was dried and dissolved in 1 mL of DMSO. Once completely dissolved, samples were quantified by UV-Vis spectrophotometry at 370 nm. Experiments were performed in triplicate and under sink conditions.

The quantification of C6 encapsulated inside PCL NPs was performed using a fluorescent detection method [27]. After dissolving the pellet in 1 mL of DMSO, the resulting solution was measured using fluorescence radiation in a multi-well plate reader (excitation and emission wavelengths at 485 and 528 nm, respectively). Experiments were performed in triplicate and under sink conditions.

The experiments performed in preformulation studies make it possible to conclude that PCL and T80 did not interfere in the analysis of both C27 and C6.

### 2.5. Spectroscopic Analysis

NMR spectra of free chromone C27, PCL@C27, and PCL NPs were acquired on a Bruker AMX 300 spectrometer operating at 400.13 MHz, at ambient temperature. Samples were dissolved in CDCl_3_ and analyzed with a final concentration of 30 mg/mL. Chemical shifts are quoted in *δ* (ppm) values relative to tetramethylsilane (TMS) used as internal reference. Coupling constants (*J*) are given in Hz.

### 2.6. Particle Size, Zeta Potential, and Morphology Analysis

The hydrodynamic particle size (D*_DLS_*), polydispersity index (PdI), and zeta potential (z-potential) of all prepared NPs were analyzed by dynamic light scattering (DLS) and electrophoretic mobility, using a Zetasizer (Litesizer™500, Anton Paar, Graz, Austria) equipped with a 4.0 mW internal laser. The resulting concentrated solutions of different NPs formulations were diluted (10% *v*/*v*) in Milli-Q water, phosphate buffered saline solution PBS (1×), and Hank’s balanced salt solution, with calcium and magnesium HBSS (+/+). Each value resulted from triplicate determinations.

The morphology of the PCL and PCL@C27 NPs were analyzed by scanning electron microscopy (SEM) (JEOL JSM-6390, Tokyo, Japan). The experimental details were described in [8] and are detailed in the Supplementary Materials.

### 2.7. Differential Scanning Calorimetry and Powder X-ray Diffraction

Thermal analyses of chromone C27, PCL, and PCL@C27 NPs were performed in accordance with [8] and are detailed in the Supplementary Materials.

Powder X-ray diffraction (PXRD) was performed on an Empyrean PANalytical Diffractometer (CuK_α,1,2_ X-radiation; λ_1_ = 1.540598 Å; λ_2_ = 1.544426 Å), equipped with a PIXcel 1D detector and a flat-plate sample holder in a Bragg–Brentano para-focusing optics configuration (45 kV, 40 mA) (Panalytical, Almelo, The Netherlands). Data were collected at ambient temperature. Intensity data was measured by the step-counting method (step 0.01°), in continuous mode, over the scan range (2*θ*) between 3.5 and 50°.

### 2.8. In Vitro Release Studies

The in vitro release studies were carried out using PBS (1×, pH 7.4) or a sequential combination of PBS (1×, pH 7.4) followed by HCl (0.1 N, pH 1.2), to mimic the gastrointestinal track as release mediums [8]. Briefly, 1 mL of concentrated PCL@C27 nanoformulation was centrifuged (10 min, 16,060× *g*, 4 °C) to separate the supernatant from the nanoformulation. The resulting pellet was resuspended in 1 mL of release medium. The eppendorfs (1.5 mL) were kept in a bath-shaker at 37 °C and 90 rpm. At predetermined time intervals, the nanosuspension was centrifuged (10 min, 16,060× *g*, 4 °C) and the supernatant removed and replaced by fresh release medium. All collected supernatants were stored at 4 °C until quantification. Experiments were performed in triplicate and under sink conditions. 

### 2.9. In Vitro Cellular Studies

#### 2.9.1. Cell Lines and Culture Conditions

Human neuroblastoma differentiated cells (SH-SY5Y cell line), epithelial colorectal adenocarcinoma cells (Caco-2 cell line), and cerebral microvascular endothelial cells (hCMEC/D3 cell line) were used as in vitro models. Details on the culture conditions are described in the literature [8] and in the Supplementary Materials.

#### 2.9.2. Cell Viability Assays

After the confluence was reached, SH-SY5Y, Caco-2 and *h*CMEC/D3 cells were exposed to different concentrations of the chromone under study (2.5, 5.0, and 10.0 μM) and PCL NPs (25–100 μg/mL of nanoformulation powder) in fresh cell culture medium. The cell viability was evaluated using the 3-(4,5-dimethylthiazol-2-yl)-2,5-diphenyltetrazolium bromide (MTT) reduction assay [28]. After 24 h of exposure (SH-SY5Y and Caco-2 cells), or 5 h in the case of *h*CMEC/D3 cells, the cell culture medium was aspirated, and fresh cell culture medium containing 0.5 mg/mL MTT was added. Afterwards, SH-SY5Y and Caco-2 cells were incubated for 1 h and *h*CMEC/D3 cells were incubated for 4 h, at 37 °C, in a humidified 5% CO_2_–95% air atmosphere. Then, the cell culture medium was removed, and the formed formazan crystals dissolved in 100% DMSO. The absorbance was measured at 550 nm in a multi-well plate reader (PowerWaveX BioTek Instruments, Winooski, VT, USA). The results are expressed as a percentage of the control (nontreated) of three independent experiments (performed in triplicate).

#### 2.9.3. Cellular Uptake and Intracellular Localization Studies

The capacity of PCL NPs to be internalized in SH-SY5Y and *h*CMEC/D3 cells was determined using a fluorescent probe (coumarin C6) encapsulated inside PCL NPs (PCL@C6 NPs) and measured by fluorescence spectroscopy. After incubation with PCL@C6 NPs at 50 and 100 μg/mL (conditions described in cell viability analysis section), cells were washed thrice with fresh HBSS (+/+) to remove the excess of PCL@C6 NPs. Then, HBSS (+/+) was added and the intracellular fluorescence assessed in a multi-well plate reader (excitation and emission wavelengths at 485 and 528 nm, respectively). For the intracellular localization, after treatment with PCL@C6 NPs (50 μg/mL), cells were washed thrice with fresh HBSS (+/+) and treated with 5 µg/mL of Hoechst 33,342 for 30 min at 37 °C in the dark. Afterwards, the cells were washed twice with fresh HBSS (+/+) and images were captured with an automated microscope (Lionheart FX, BioTek, Winooski, VT, USA). After this, the images were superimposed to determine the intracellular localization of the NPs.

#### 2.9.4. Evaluation of MAO-B Activity by Fluorescence Kynuramine Assay

The capacity of C27 and nanoformulations to inhibit MAO-B in a cell SH-SY5Y-based model was performed, as described by Santillo et al., with minor modifications [29]. Briefly, after a prior inhibition of MAO-A with a standard MAO-A inhibitor (clorgyline), the activity of MAO-B was measured by the metabolization of kynuramine to 4-hydroxyquinoline, which presents fluorescence properties. After the confluence was reached, SH-SY5Y cells were incubated with clorgyline (100 nM) for 30 min. Then, cells were co-incubated with kynuramine (60 µM) and test compounds (C_C27_ = 1 µM) for 8 h at 37 °C. The reaction was stopped by the addition of NaOH (0.5 M) to the well and the fluorescence was measured in a multi-well plate reader (excitation and emission wavelengths at 360 and 460 nm, respectively). Selegiline (100 nM) was used as a standard inhibitor of MAO-B and cells without treatment and treated only with kynuramine were used as controls. The results are expressed as a percentage of the control (nontreated), normalized with the protein content of the three independent experiments (performed in triplicate).

#### 2.9.5. Protein Quantification

The activity of MAO-B was normalized to the protein content of the cell lysate, which was determined by a bicinchoninic acid (BCA) assay, as described by other authors [8], using bovine serum albumin as a standard.

#### 2.9.6. Cellular Permeability Studies 

The permeability of PCL@C6 NPs in human intestinal epithelium was evaluated using Caco-2 cells monolayer as an in vitro model [30]. The experimental details were performed as described in [8] and are presented in the Supplementary Materials.

#### 2.9.7. Rhodamine 123 Accumulation Assay

The P-glycoprotein (P-gp) inhibitory activity of both C27 and PCL@C27 NPs was determined by measuring the intracellular accumulation of rhodamine (RHO) 123 in Caco-2 and *h*CMEC/D3 cells, in the absence or presence of P-gp inhibitors, as described in the literature [31]. Briefly, after reaching the confluence, cells were pre-treated with C27 (10 μM) and PCL@C27 NPs (100 μg/mL) for 30 min. Then, 20 µM RHO 123 was added and the cells were incubated for 90 min at 37 °C. Elacridar, a potent third-generation P-gp inhibitor (10 µM), was used as a positive control. After this time, cells were washed with PBS, lysed with DMSO, and the intracellular levels of RHO 123 were quantified by fluorimetry using a multi-well plate reader (PowerWaveX BioTek Instruments, Winooski, VT, USA) (excitation and emission wavelengths were 485 and 535 nm, respectively). Data was expressed as the percentage of RHO 123 accumulation relative to control cells (untreated with P-gp inhibitors), arbitrarily set at 100%.

### 2.10. Statistical Analysis

Physicochemical and in vitro data are presented as the mean ± standard deviation (SD). Data analysis for all the studies are specified in the Supplementary Materials.

## 3. Results

### 3.1. Preparation and Characterization of PEGylated PCL-Based Nanoformulations

The encapsulation of chromone C27 inside PEGylated polycaprolactone nanoparticles (PCL NPs) was successfully obtained by the nanoprecipitation method using polysorbate 80 (Tween^®^ 80, T80) as a stabilizer agent. The first step was to establish the C27 optimal concentration to be used during the encapsulation process, being PCL NPs fed with different chromone C27 amounts (2.5, 5 and 10% of PCL, *w*/*w*). It is important to note that feeding PCL NPs with amounts of C27 higher than 10% caused the destabilization of the nanoformulation, with the concomitant formation of aggregates (data not shown). After complete evaporation of the acetone, all stable nanoformulations were ultrafiltrated to remove any trace of free chromone C27 and also of T80 that were not adsorbed to the PCL NPs surface. 

**Figure 2:**
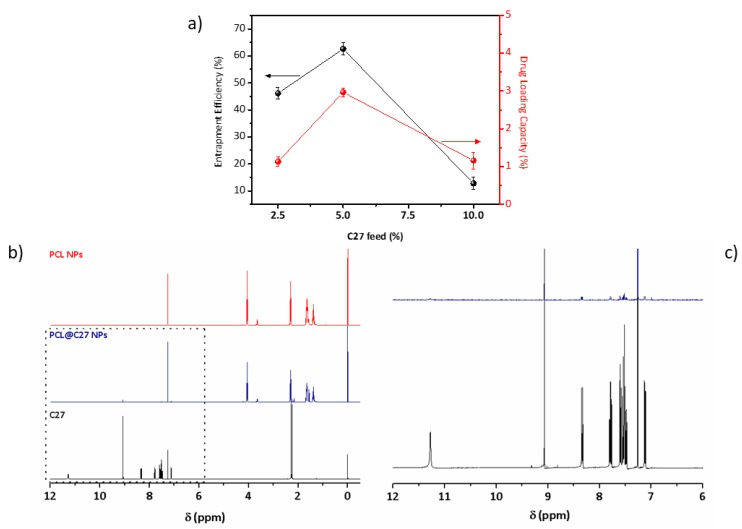
Physicochemical characterization of PCL@C27 nanoformulation by (**a**) determination of the encapsulation efficiency (EE%) and drug loading capacity (DLC%) of NPs fed with different amounts of chromone C27 (2.5, 5, and 10%). (**b**) Structural characterization of C27, PCL NPs, and PCL@C27 NPs using ^1^H NMR between 0 and 12 ppm, and (**c**) respective magnification between 12 and 6 ppm of C27 ^1^H NMR spectra. Values of entrapment efficiency (EE%, black) and drug loading capacity (DLC%, red) obtained for PCL@C27 NPs prepared with different amounts of feeding C27. Measurements of C27 quantification were performed in triplicate and results are presented as mean ± SD.

The resulting PCL@C27 nanoparticles were analyzed and the data of encapsulation efficiency (EE%) and drug loading capacity (DLC%) determinations are summarized in Figure 2a (detailed information in the Supplementary Materials). 

As observed in Figure 2a, the highest EE% (62.5 ± 2.3%) and DLC% (2.96 ± 0.12%) values were obtained for the NPs with a 5% C27:PCL ratio. As a result, under these conditions, the final C27 concentration (C_C27_) value of PCL@C27 NPs was 263.3 ± 12.1 µM, presenting a concentration approximately 400,000-fold higher than the concentration required for the therapeutic effect (MAO-B IC_50_ = 670 ± 130 *p*M). 

To confirm the PCL NPs structural composition ^1^H NMR spectra were acquired (Figure 2b). The ^1^H NMR spectra of the nanoformulations (red and blue lines) showed the PCL characteristic peak resonances at 1.38, 1.65, 2.30, and 4.06 ppm, which are related to the CH_2_ protons of the PCL backbone [32]. Also, the peak located at 3.63 ppm, assigned to a CH_2_, corroborates the presence of T80 in both PCL NPs [33]. In addition, in the PCL@C27 NPs spectra (blue line), the signals caused by the presence of the benzopyrone proton peaks were observed at 2.25 and 2.28 ppm, which are related to the two methyl groups located on the exocyclic aromatic ring, and between 9.1–7.1 ppm. The data confirm the presence of the C27 chromone in the NPs. The detailed C27 ^1^H NMR data (black line) was included in the Supplementary Materials.

### 3.2. Differential Scanning Calorimetry and Powder X-ray Diffraction Analysis

**Figure 3:**
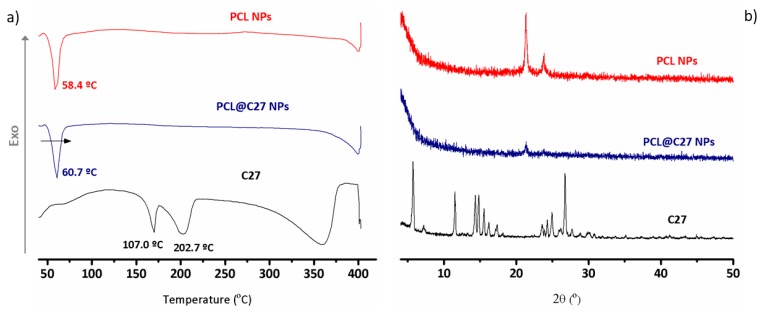
Physicochemical characterization of chromone C27, PCL NPs, and PCL@C27 NPs using (**a**) differential scanning calorimetry (DSC) between 40 and 400 °C, and (**b**) powder X-ray diffraction (PXRD) in the range of 4–50 °C.

Chromone C27, unloaded, and loaded PCL NPs were subjected to thermal analysis to obtain information regarding their crystalline morphology, as well as information on putative interactions between C27 and the PCL polymeric matrix [34,35]. The differential scanning calorimetry (DSC) curves are depicted in Figure 3a. 

For chromone C27 (black curve) a typical thermogram of a crystalline structure with two endothermic peaks at 170.0 °C and 202.7 °C was obtained. Since chromone C27 thermogravimetric analysis (data not shown) did not show any weight loss in this range of temperatures, these peaks were not due to evaporation of water. Instead, the first peak could be related to a glass transition process [36] and the second peak to the C27 melting transition, characterized by a *T*_max_ = 233–236 °C, which is approximately the same value of the C27 melting point obtained by capillary method (Supplementary Materials).

As none of these peaks appeared in the PCL@C27 NPs DSC curve (in blue), it can be concluded that C27 is encapsulated, probably in an amorphous or disordered phase [37]. The melting endothermic peak at 58.4 °C presented by the PCL NPs (in red) suffered a shift of 2 °C in the PCL@C27 NPs (60.7 °C, Figure 3a), a change that discloses a strong interaction between C27 and the PCL matrix [8,38]. 

As the crystallinity of the encapsulated C27 into PCL NPs can influence the drug release properties of the nanoparticles [39], powder X-ray diffraction (PXRD) data were acquired. 

The diffractograms of C27, unloaded, and loaded NPs are depicted in Figure 3b. The diffractogram of C27 exhibited sharp peaks that are intrinsically related to the crystalline nature of chromone. For nanoformulations, a semi-crystalline structure with two sharp peaks between 20 and 25° was detected [40]. Moreover, a decrease in polymer crystallinity was observed when comparing both PCL and PCL@C27 nanoformulations, which can be related to an interaction between the chromone and the polymer. The diffractogram of PCL@C27 NPs does not have any reflection pertaining to C27, which suggests its complete amorphization when loaded in the polymeric matrix [41]. These results are in good agreement with the literature [42]. Moreover, the data corroborate well with the results of DSC analysis.

### 3.3. PEGylated PCL@C27 Particle Size, z-Potential, and Morphology

**Figure 4:**
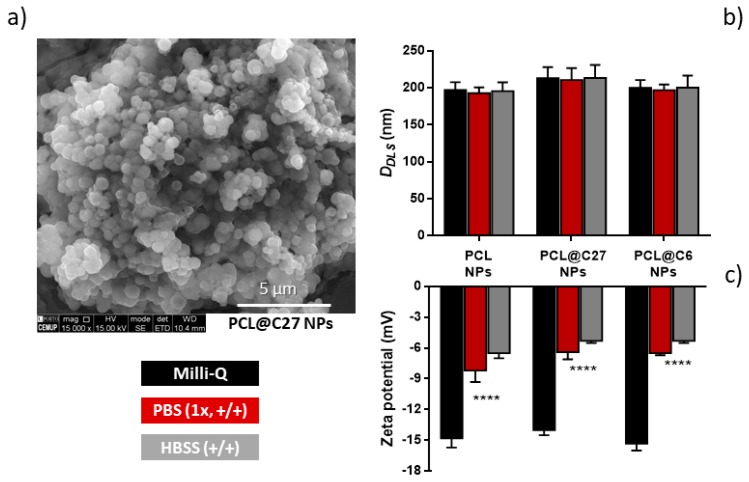
Morphological characterization of PCL@C27 NPs by scanning electron microscopy (SEM) (**a**) and hydrodynamic average sizes (D*_DLS_*) (**b**) and zeta potential values (**c**) of PEGylated PCL NPs in Milli-Q water, phosphate buffered saline solution (PBS) (1×, +/+), and Hank’s balanced salt solution (HBSS) (+/+), as measured by dynamic light scattering (DLS). All measurements were performed in triplicate and results are given as mean ± SD. Statistical comparisons were made using two-way ANOVA. In all cases, *p*-values lower than 0.05 were considered significant (**** *p* < 0.0001 versus Milli-Q water values).

The morphology and shape of PCL@C27 NPs were evaluated using scanning electron microscopy (SEM, Figure 4a). 

The PCL@C27 nanoformulation presented NPs with a spherical shape and a uniform size distribution (Figure 4a), although some aggregation, probably due to the drying process, was observed. The hydrodynamic size (D*_DLS_*) and zeta potential (z-potential) of C27-loaded and unloaded PEGylated PCL NPs were assessed in three different conditions: Milli-Q water, phosphate-buffered solution with calcium and magnesium (PBS 1×, +/+), and Hank’s balanced salt solution with calcium and magnesium (HBSS +/+) (Figure 4b,c). Although particle characterization measurements are commonly conducted in Milli-Q water [43], the morphology and surface charge of NPs were also evaluated in PBS and HBSS, the mediums used in drug-controlled release and cell-based studies, respectively.

Since PEGylated PCL NPs containing a fluorescent coumarin-based probe (PCL@C6 NPs) were used for the cellular uptake, intracellular localization, and permeability studies, it was necessary to compare the morphology between both PCL@C27 and PCL@C6 nanoformulations.

For all tested media, the nanoformulations presented monodisperse profiles with D*_DLS_* lower than 250 nm (Figure 4b). In fact, in physiological mediums (PBS and HBSS medium), PCL@C27 NPs had D*_DLS_* values between 211 and 213 nm. As NPs sized circa 200 nm have been reported to be able to cross biological barriers, by preventing spleen filtration and reducing the opsonization by reticuloendothelial system, this is considered an encouraging result [44,45]. Despite no significant morphological differences being observed, the presence of chromone C27 seemed to influence the size of PCL NPs in both media, as they presented a slightly larger size when compared to unloaded NPs (~3–7% higher size values). This data is in good agreement with the literature [25,46]. 

The stability of NPs in aqueous medium is often assured by the presence of a surface charge, as it avoids the aggregation process. Without surfactant, PCL NPs usually present a z-potential between −35 and −30 mV in Milli-Q water, due to the negatively charged ionized carboxylic acid groups of the polymer [37]. In our case, the presence of T80 in NPs surface led to a reduction of the z-potential value to −14.0 and −15.3 mV in Milli-Q water for PCL NPs and PCL@C27 (Figure 4c), respectively. In physiological medium, the z-potential values (between −5.3 and −8.2 mV) were significantly different (*p* < 0.0001) from those obtained in Milli-Q water. This data is in accordance with what has been previously reported [8], and can be ascribed to the presence of interactions of opposite charged ions with the NPs surface [47,48]. The presence of T80 and negative charge in NPs surface could justify the high storage stability at 4 °C over three months, since both D*_DLS_* and z-potential NPs remained unchanged and no aggregates were observed (data not shown).

The data showed non-significant differences in terms of NPs size and surface charge density, when comparing PCL@C6 to PCL@C27 NPs (Figure 4b,c). These results allow us to use the nanoformulation PCL@C6 as a model of C27 delivery carrier in cellular studies.

### 3.4. In Vitro C27 Release Kinetics

**Figure 5:**
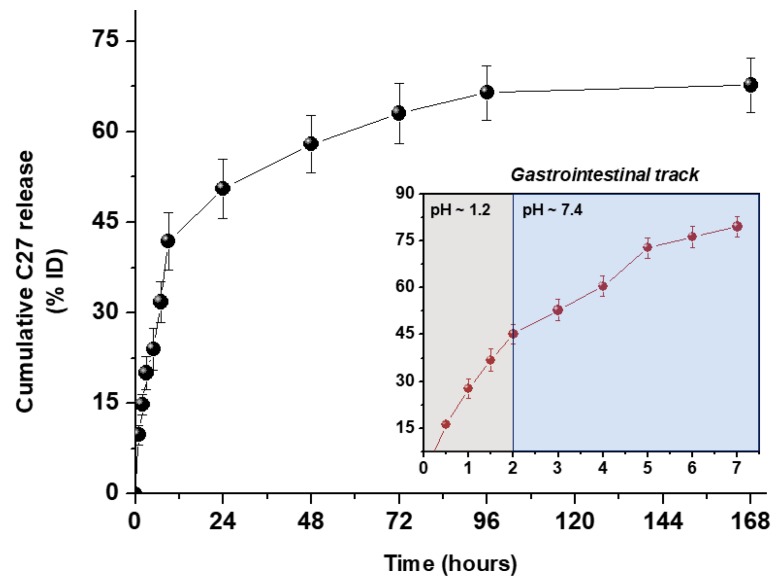
In vitro release profile of chromone C27 from PCL@C27 NPs in PBS (pH 7.4) conducted for seven days (black dot). Inset: In vitro release profile in 0.1 N HCl, pH 1.2, for 2 h followed by PBS, pH 7.4, for 5 h (red data). Results are presented as means ± SD of three independent experiments.

The evaluation of C27 sustainable release from PCL@C27 NPs was performed in PBS (pH 7.4) at 37 °C for seven days, and at pH 1.2 for 2 h, followed by pH 7.4 for 5 h, to simulate the passage through the upper human gastrointestinal tract [49]. In both conditions, the in vitro release profile from PCL@C27 NPs was obtained by graphing the cumulative percentage of the released C27 with respect to the amount of chromone encapsulated as a function of the time (Figure 5).

The in vitro release profile showed a typical biphasic pattern, with an initial burst release in the first 9 h, followed by a slow and continuous release up to 168 h. The initial burst estimated for PCL@C27 NPs was 41.8 ± 4.8% and can be related to the dissolution in the release media of C27 chromone adsorbed in the NPs surface [19]. After the initial burst, C27 was slowly released from NPs until the end of the experiment (seven days), reaching a plateau with a total C27 cumulative release amount of 67.7 ± 4.4%. The final C_C27_ in release medium was 174.2 ± 11.5 µM (approximately 275,000-fold higher than C27 MAO-B IC_50_ value). The second phase of the biphasic release pattern is assumed to be related to: (1) a slow C27 diffusion from the PCL matrix, (2) a progressive degradation/erosion of the polymeric matrix in physiological medium, or (3) a mixed course involving both processes [50]. 

The in vitro release studies in gastrointestinal simulated fluids (inset of Figure 5) make it possible to conclude that the C27 release is accelerated in acidic medium, in a process probably related to a faster degradation of PCL. Comparing the data obtained in both media in the first 2 and 7 h, a circa 3- and 2.5-fold increase of C27 release was observed in the acidic medium, respectively. This data is in good agreement with data previously reported by our group related to the release of a coumarin-based compound from PEGylated PLGA NPs [8].

The data obtained from in vitro drug release studies was fitted to the Korsmeyer–Peppas model [12] and the results are summarized in Table 1. 

The regression coefficient (R^2^) of the plot of log *Mt*/*M∞* versus log *t* for NPs in PBS (1×) was found to be 0.972, with values of the release exponent (*n*) and release constant (K) of 0.464 and 11.2, respectively. In the case of the use of a combined acidic and PBS medium (R^2^ = 0.985), the release exponent and release constants values were 0.594 and 26.9, respectively. To sum up, the type of medium affected the way and rate of C27 release: (a) when PBS was used as medium, the *n* value was <0.5, showing that the release of C27 from NPs can be explained by Fickian diffusion [12]; (b) when an acidic medium was employed, the *n* value was higher than 0.5, suggesting a release process controlled by polymer erosion [51]. This assumption was corroborated by the higher release constant (26.9 h^−1^) in the first 2 h of the experiment.

In conclusion, C27 was successfully released from PCL NPs, with good yields and in a sustained way, regardless of its low solubility profile and strong interaction with the PCL matrix.

### 3.5. In Vitro Cellular Studies

#### 3.5.1. In Vitro Cytotoxicity in Neuronal, Intestinal, and Endothelial Cells 

Since IMAO-B operate by metabolic inactivation, and thus increase dopamine activity in the neuron synaptic cleft and at respective postsynaptic receptor sites [52], a human model of neuronal cells was chosen (differentiated neuroblastoma SH-SY5Y cells) to evaluate the cytotoxic profile of free C27 and PCL@C27 NPs. The neuroblastoma SH-SY5Y cell line is widely used in studies requiring neuronal-like cells, as they express a number of dopaminergic neuronal markers [53,54]. Furthermore, the cytotoxic effects of chromone C27 and PCL@C27 NPs were also evaluated in epithelial colorectal adenocarcinoma (Caco-2) and in human brain microvascular endothelial (*h*CMEC/D3) cells, as they are widely used in vitro models to determine the intestinal and blood-brain barrier (BBB) permeability, respectively [25,30]. 

**Figure 6:**
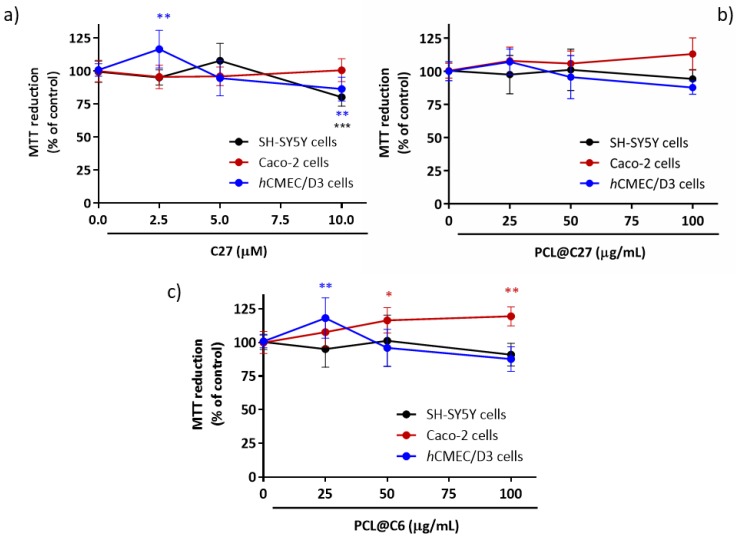
Cytotoxicity profile of C27 at 2.5, 5, and 10 µM (**a**) as well as nanoformulations of PCL@C27 (**b**) and PCL@C6 NPs (**c**) at 25, 50, and 100 µg/mL in different cells lines, evaluated by the MTT reduction assay 24 h after exposure. Results are expressed as mean percentage of MTT reduction ± SD of three independent experiments. In all cases, *p*-values lower than 0.05 were considered significant (* *p* < 0.05, ** *p* < 0.01, *** *p* < 0.001 versus control cells).

The cellular viability of C27 (2.5, 5.0, and 10.0 μM) and PCL@C27 NPs (25–100 μg/mL of nanoformulation powder) was indirectly determined by the measurement of the cellular metabolic activity using the MTT reduction method, 24 h after exposure (Figure 6). The PCL@C27 NPs concentration range was chosen in order to test nanoformulation at same C27 concentration as free C27 and taking into account the final C_C27_ (263.3 ± 12.1 µM). Furthermore, an end-point of 24 h was performed to avoid possible interferences of cytotoxic effects of free C27, since, as observed in controlled release data (Figure 5), after this period of time, around 50% of C27 is released from NPs. 

No significant cytotoxic effects were observed in Caco-2 cells when exposed to C27, when compared to control cells, and for the conditions tested (Figure 6a). On the contrary, a slight but significant decrease in the metabolic activity was observed when SH-SY5Y (80.3 ± 2.4%, *p* < 0.01) and *h*CMEC/D3 cells (86.5 ± 3.2%, *p* < 0.05) were exposed to C27 at the highest concentration (10 µM) for 24 h. 

In the case of PCL@C27 NPs, in the present experimental conditions, no significant cytotoxic effects were noticed (Figure 6b), even for the highest concentration tested (100 µg/mL), which corresponded to a final encapsulated C27 concentration of around 10.6 µM. 

Although efforts were made to evaluate the uptake and permeability of chromone C27 and PCL@C27 NPs across SH-SY5Y, Caco-2, and *h*CMEC/D3 cells, these studies were hampered by the chromone spectral features and the sensitivity of the analytical method. Therefore, a model probe was used to validate the carrier properties of the PCL nanoformulation. As previously mentioned, a probe based on coumarin scaffold (coumarin C6, Figure 1) [55], a benzopyran analogue, was chosen to attain the goal, due to its structural similarity and unique fluorescent properties. After the preparation and morphologic characterization of PCL@C6 NPs, the cytotoxic profile of the PCL@C6 nanoformulation was evaluated using the same conditions described for the PCL@C27 NPs studies. For all tested cell lines, no significant reduction in the metabolic activity was detected, for concentrations up to 100 µg/mL, and after 24 h of incubation.

Summing up the data herein obtained reinforced the benefit of using PEGylated PCL NPs as carriers of bioactive compounds that present bioavailability drawbacks.

#### 3.5.2. In Vitro Cellular Uptake and Intracellular Localization in Neuronal and Endothelial Cells

**Figure 7:**
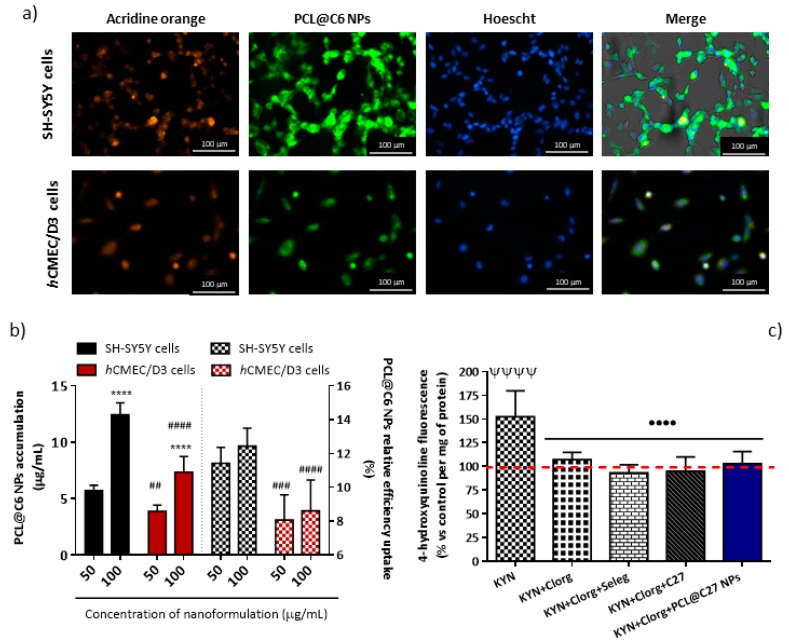
Representative images of acridine-orange and Hoescht stained SH-SY5Y and *h*CMEC/D3 cells treated with PCL@C6 NPs at 50 µg/mL for 24 and 5 h, respectively (**a**); accumulation (solid bars) and relative efficiency uptake (patterned bars) of PCL@C6 NPs (50 and 100 µg/mL) in SH-SY5Y and *h*CMEC/D3 cells after 24 and 5 h of exposure, respectively (**b**); evaluation of the MAO-B inhibitory capacity in SH-SY5Y cells after 8 h of exposure, using kynuramine (KYN) as the substrate and clorgyline (Clorg) and selegiline (Seleg) as standard inhibitors of MAO-A and MAO-B, respectively (**c**). The results are expressed as mean ± SD (*n* = 3) and statistical comparisons were made using two-way ANOVA and one-way ANOVA, in cases (**b**) and (**c**), respectively. In all cases, *p*-values lower than 0.05 were considered significant (**** *p* < 0.0001 by comparison of different concentrations tested concentration; ^##^ *p* < 0.01, ^###^ *p* < 0.001, and ^####^ *p* < 0.0001 by comparison the results obtained between the two cell lines tested; ^ΨΨΨΨ^ *p* < 0.0001 by comparison with untreated cells; ^••••^ *p* < 0.0001 by comparison with cells treated only with kynuramine). The dashed red line represents the control data.

The intracellular localization of PEGylated PCL NPs was verified by fluorescence microscopy in neuronal and endothelial (SH-SY5Y and *h*CMEC/D3) cells that express MAO-B [56,57]. For this, cells were exposed to PCL@C6 NPs (50 µg/mL) for 24 and 5 h, respectively. After that, they were washed with HBSS (+/+) to remove the excess PEGylated PCL NPs and the cell’s nucleus was marked with Hoechst 33,342 (blue stain) and the nucleic acid (either DNA or RNA) with acridine orange (orange stain). From the data (Figure 7a), it was observed that PCL@C6 NPs (green stain) were dispersed in the cytoplasm of the cells.

As described above, after the exposure to PCL@C6 NPs at 50 and 100 µg/mL, cells were washed with HBSS (+/+) and the remaining fluorescence was measured by exciting the media with a radiation of 485 nm. The results are presented as the intracellular accumulation and relative efficiency uptake of PCL@C6 NPs (50 and 100 µg/mL, Figure 7b). 

The cellular uptake of PCL@C6 NPs is supposed to occur via endocytosis [58], and in both cell lines is significantly different and influenced by the initial concentration (IC) used in the treatment of the cells (Figure 7a). For the same concentration, PCL@C6 NPs uptake is higher in neuronal cells than in endothelial cells (Figure 7a, solid bars). Actually, a proportionality between the concentration and the uptake was established: the exposure to PCL@C6 NPs at 100 µg/mL resulted in a 2.2- and 1.9-fold higher cellular uptake (**** *p* < 0.0001, Figure 7a, solid bars) in neuronal and endothelial cells, respectively, when compared to the 50 µg/mL concentration. However, after comparing the efficiency of cellular uptake for the concentrations tested (50 and 100 µg/mL), no significant difference was observed (Figure 7a, patterned bars).

Since the structure and morphology of PCL@C27 and PCL@C6 NPs are similar, we suggested that both nanoformulations presented identical cellular uptake profiles, which allowed us to correlate the concentrations of nanoformulations PCL@C6 and PCL@C27 inside the cells. After the end-points, minimum nanoformulation concentrations of 5.7 ± 0.2 and 3.9 ± 0.2 µg/mL were determined for SH-SY5Y and *h*CMEC/D3 cells (Figure 7a, solid bars), respectively, which corresponded to a final C27 concentration between 410 and 606 nM. This range of concentration is 651- to 963-fold higher than the C27 MAO-B IC_50_ value. 

To demonstrate the MAO-B inhibitory activity of the systems in a cell-based model (SH-SY5Y), additional studies were performed in the presence and absence of C27 and nanoformulation PCL@C27 [29]. The activity of MAO-A was inhibited by pre-treatment with clorgyline (100 nM) and the fluorescence intensity was normalized to the protein content. The same procedure was followed with the MAO-B inhibitor selegiline (100 nM).

After 8 h of experiments, and in the absence of MAO inhibitors, an increment of fluorescence intensity caused by the metabolization of kynuramine to 4-hydroxyquinoline (fluorescent metabolite) was observed, when compared with untreated cells (^ΨΨΨΨ^ *p* < 0.0001, Figure 7c). Meanwhile, when both MAO-A and MAO-B inhibitors were used, a reduction in the intensity of fluorescence was observed, when compared with cells treated only with kynuramine (^••••^ *p* < 0.0001, Figure 7c). The same protocol was applied with selegiline. In this case, a slight decrease in fluorescence, when compared with the treatment done only with clorgyline, was detected. This effect is related to the inhibition of both MAO isoforms. Since the treatment with C27 and PCL@C27 NPs showed the same tendency as the data obtained with selegiline, it was concluded that C27 works as a MAO-B inhibitor in neuronal cells. 

The combined data of microscopy analysis, cellular uptake, and MAO-B activity in cell-based models suggested that PEGylated PCL NPs can deliver C27 into the cellular cytoplasmatic matrix. Even further, it was shown that C27 and PCL@C27 NPs are capable of inhibiting MAO-B in neuronal cells.

#### 3.5.3. In Vitro Permeability Studies in Epithelial and Endothelial Cells

Several in vitro BBB models, also used by the (bio)pharma industry, have been developed to assist the selection and preclinical evaluation of CNS drug candidates [59]. Among those based on primary cultures of cerebral endothelial cells or immortalized cell lines, the human brain endothelial cell line (*h*CMEC/D3) is one of the most studied in drug transport and uptake experiments [60]. However, other models based on epithelial cell lines have been also used to predict the permeability of CNS drug candidates, due to their advantages in terms of costs [59,60]. In fact, the Caco-2 human intestinal epithelial cell line is one of the most widely in vitro models used to predict human drug absorption, mainly for small intestine absorption [61]. 

Accordingly, the ability of PEGylated PCL NPs to cross biological barriers was evaluated in two different cell lines (Caco-2 and *h*CMEC/D3 cells) [8]. These cell lines retain the expression of most transporters and receptors expressed in vivo in the human intestine and BBBs and have been widely used in several permeability studies related to Alzheimer’s and Parkinson’s diseases [8,62,63]. Both cell lines were cultured in a cell culture insert filter (Transwell) and used after obtaining cellular monolayers with high integrity, a parameter that was verified by the measurement of transendothelial electrical resistance values. The number of PCL@C6 NPs accumulated in basal medium was normalized with the weight of protein presented in the respective Transwell. 

**Figure 8:**
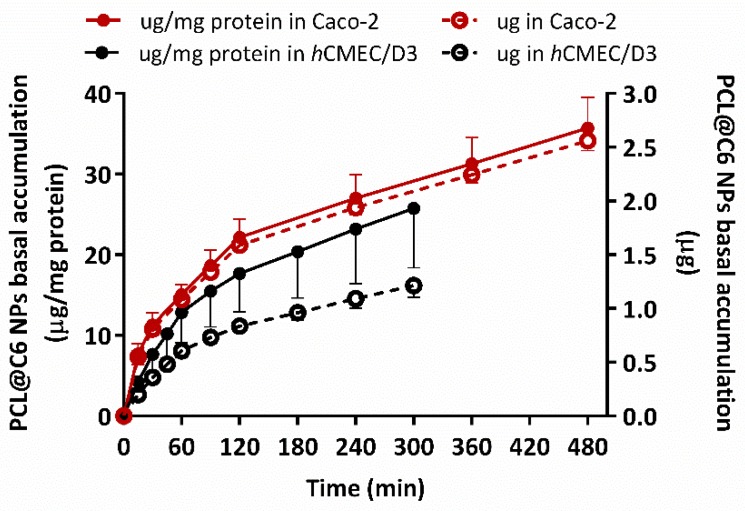
Amount of PCL@C6 NPs, with (µg/mg of protein, solid circles and line) or without (µg, empty circles points and dash line) protein mass normalization, accumulated in basal medium after 5 h and 8 h of permeability experiment in *h*CMEC/D3 (black data) and Caco-2 (red data) cells with a 100 µg/mL initial concentration. Results are expressed as mean ± SD of three independent experiments.

The data presented in Figure 8 show that PCL@C6 NPs (100 µg/mL) exhibited two permeability profiles with a biphasic tendency, which can be ascribed to a mixed passive and transport-mediated mechanism [8,64]. Although, in terms of the particle size, the transport mechanism established for particles up to 200 nm is the receptor-mediate endocytosis [65], the use of T80 as a coating agent in PCL NPs could enhance the paracellular permeation in epithelial and endothelial cell monolayers [66,67]. In fact, it was demonstrated in vivo that the accumulation of donepezil in the brain was significantly higher when the drug was encapsulated in PLGA NPs, and that Tween 80 can improve the opening of the tight junctions at the BBB and inhibit the P-glycoprotein efflux system [68]. 

In Caco-2 cell monolayers, a final accumulation of 35.7 ± 3.8 µg/mg protein (solid red circles), were obtained with an apparent permeability coefficient (P_app_) value calculated of 4.7 × 10^−7^ cm/s [69]. Meanwhile, after 5 h of experiment, a final PEGylated PCL NPs concentration of 25.7 ± 7.4 µg/mg protein (solid black circles) was found in basal medium, with a concomitant P_app_ value of 1.2 × 10^−7^ cm/s [69]. The combined data showed that PEGylated PCL NPs were capable to cross both intestinal and BBB barriers in a time-dependent manner.

In terms of nanoformulation mass (empty circles, Figure 8), 2.56 ± 0.09 (red empty circles, Figure 8) and 1.21 ± 0.11 µg (black empty circles, Figure 8) were detected at the end of experiment, in the basal medium of Caco-2 and *h*CMEC/D3 cells, respectively. The slight differences between the values obtained in permeability assays could be associated with the type of cells and intrinsic mechanisms of passive transport [70]. 

In line with the results of cellular uptake, we hypothesize that PCL@C27 nanoformulation is also able to cross intestine and BBB in vitro monolayers in the same extension as PCL@C6 NPs. Thus, extrapolating the results obtained for PCL@C6 NPs, a final C27 concentration of 117.7 and 55.8 nM (at least one order of magnitude higher than C27 MAO-B IC_50_ value) could be expected.

PEGylated PCL NPs showed the ability to circumvent in vitro BBB cell monolayer hindrances and deliver an IMAO-B at concentrations higher enough to induce a therapeutic effect.

#### 3.5.4. Rhodamine 123 Accumulation in Epithelial and Endothelial Cells

The inhibition of efflux pumps is described to be a parameter of utmost importance in drug delivery [71]. In fact, after the discovery that polymeric pharmaceutical excipients can inhibit efflux pumps, various other polymers have been studied regarding their potential efflux pump inhibitory activity [72]. Excipients containing polyethylene glycol and PEGylated surfactants, such T80 or poloxamers, have been described to efficiently inhibit efflux pumps, namely P-glycoprotein (P-gp) [73,74,75]. 

**Figure 9:**
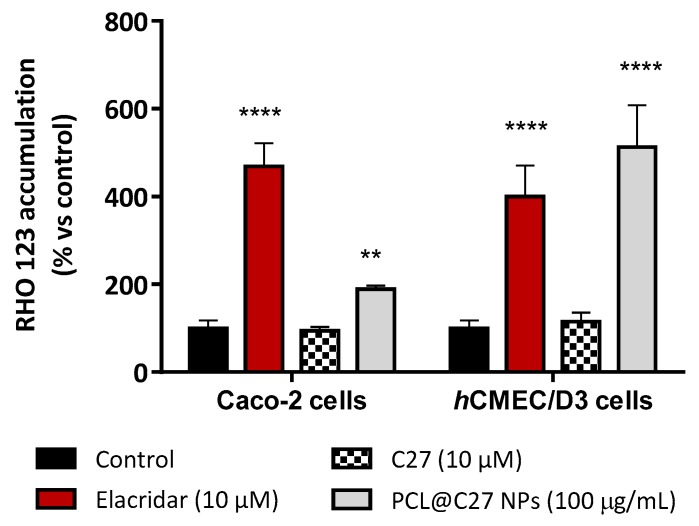
Accumulation of Rhodamine (RHO) 123 in Caco-2 and hCMEC/D3 cells. Data are expressed as percentage of fluorescence dye accumulation in control cells exposed only to RHO 123, arbitrarily set as 100%, and are the means ± SD of three independent assays. In all cases, *p*-values lower than 0.05 were considered significant (** *p* < 0.01 and **** *p* < 0.0001).

In this work, Caco-2 and *h*CMEC/D3 cells were used as in vitro models and the P-gp activity assessed using RHO 123 as a P-gp fluorescent substrate. Therefore, a decrease in P-gp activity results in a decreased amount of RHO 123 effluxed by this pump, which is followed by an increase in the intracellular fluorescence intensity (increased Rho 123 intracellular content). For that purpose, cells were pre-treated for 30 min with C27 (10 µM), PCL@C27 NPs (100 μg/mL) or with elacridar (10 μM), a well-known third-generation P-gp inhibitor, used as a positive control for this experiment. After that, 20 μM RHO 123 was added and cells were incubated for 90 min. The obtained results are presented in Figure 9.

As observed in Figure 9, no significant difference in RHO123 intracellular fluorescence was verified when C27 was tested, when compared to control cells. On the contrary, elacridar (10 µM) acted as a P-gp inhibitor, avoiding the RHO 123 efflux from both cellular models, which resulted in a 5-fold increase in RHO123 intracellular fluorescence, when compared to control cells (*p* < 0.0001). The same behavior was obtained with PCL@C27 NPs. In this case, an increase of 1.9- and 5-fold of RHO 123 intracellular levels was found for the Caco-2 and *h*CMEC/D3 cells, respectively. This data is in accordance with other works, which showed that NPs coated with Tween 80 have a good capacity to inhibit P-gp [76,77]. 

## 4. Conclusions

In this work, a potent, selective, and reversible IMAO-B was successfully encapsulated in PCL NPs coated with T80. The nanoprecipitation method was employed to obtain stable particles in physiological conditions with D*_DLS_* lower than 213 nm and a z-potential of around −5 mV, with a high stability in physiological medium. The optimization process of C27 encapsulation gave rise to NPs with a final C27 concentration of 263.3 ± 12.1 µM, 400,000-fold higher than its IC_50_ value (670 ± 130 *p*M). In physiological conditions, the nanoformulation sustainably released C27 over seven days, with a final release amount of 67.7 ± 4.4%. Furthermore, no cytotoxic effects of C27 and PCL@C27 NPs were observed in Caco-2 cells. In SH-SY5Y and *h*CMEC/D3 cells, the encapsulation of C27 was essential to decrease its cytotoxicity in the conditions tested. 

The uptake and permeability studies, performed with PCL@C6 NPs, showed that PEGylated PCL NPs were capable of accumulating in the cytoplasm of both neuronal and endothelial cells and surpassing the in vitro epithelial and endothelial cell monolayers. By extrapolation, one can infer that PCL@C27 NPs are able to deliver C27 across the intestine and BBB monolayer at concentrations higher than its MAO-B IC_50_ value.

Overall, our results provide evidence of the effectiveness of PCL nanocarriers to deliver neuroactive compounds that have bioavailability and physicochemical drawbacks. Further improvement and research, focused on in vivo long-term safety and efficacy studies, on polymeric nanomedicines will be, in the future, a promising tool to boost the success of clinical trials. Given the significant costs associated with drug discovery and development, it is becoming increasingly important to engineer targeted nanomaterial carriers allowing a sustained release of new neuroactive drug candidates.

## Figures and Tables

**Table 1:** Correlation values (R^2^) and release exponent (*n*) of kinetic data analysis of chromone C27 release from PCL@C27 NPs in different medium.

Medium pH	Korsmeyer–Peppas		
	R^2^	*n*	K (h^−1^)
7.4	0.972	0.464	11.2
1.2–7.4	0.985	0.594	26.9

## Supplementary Materials

The following are available online at https://www.mdpi.com/1999-4923/11/7/331/s1. Table S1 is related to the EE% and DLC% values obtained for different formulations prepared with different amounts of feeding C27. Table S2 is related to D*_DLS_* and z-potential values obtained in Milli-Q, PBS (1×, +/+), and HBSS media (+/+). Figure S1 is related to the UV-Vis spectra of chromone C27.
