# Supplementary material for: Boosting Drug Discovery for Parkinson’s: Enhancement of the Delivery of a Monoamine Oxidase-B Inhibitor by Brain-Targeted PEGylated Polycaprolactone-Based Nanoparticles
Source: Pharmaceutics. 2019 Jul 12;11(7):331. doi: 10.3390/pharmaceutics11070331 (PMC6681091; doi:10.3390/pharmaceutics11070331)
Supplement: Supplementary file 1 [file pharmaceutics-11-00331-s001.pdf]

pharmaceutics

MDPI

Supplementary Materials: Boosting Drug Discovery for Parkinson's: Enhancement of the Delivery of a Monoamine Oxidase-B Inhibitor by Brain-Targeted PEGylated Polycaprolactone-Based Nanoparticles

Miguel Pinto [1,†], Carlos Fernandes [1,†], Eva Martins [2], Renata Silva [2], Sofia Benfeito [1], Fernando Cagide [1], Ricardo F. Mendes [3], Filipe A. Almeida Paz [3], Jorge Garrido [4], Fernando Remião [2] and Fernanda Borges [1,*]

[1] CIQUP, Departmento de Química e Bioquímica, Centro de Investigação em Química, Faculdade de Ciências, Universidade do Porto, 4169-007 Porto, Portugal
[2] UCIBIO-REQUIMTE, Laboratório de Toxicologia, Departamento de Ciências Biológicas, Faculdade de Farmácia, Universidade do Porto, 4050-313 Porto, Portugal
[3] Departamento de Química, CICECO-Instituto de Materiais de Aveiro, Universidade de Aveiro, 3810-193 Aveiro, Portugal
[4] Departamento de Engenharia Química, Instituto Superior de Engenharia do Porto (ISEP), Instituto Politécnico do Porto, 4200-072 Porto, Portugal
* Correspondence: fborges@fc.up.pt
† These authors contributed equally to this work.

Experimental Section

Synthesis of N-(3′,4′-Dimethylphenyl)-4-oxo-4H-chromene-3-carboxamide (C27)

The synthesis of *N*-(3′,4′-dimethylphenyl)-4-oxo-4H-chromene-3-carboxamide (C27) was previously reported by Reis and colleagues (Scheme S1) [1]. Briefly, to a solution of chromone-3-carboxylic acid (2.6 mmol) in DMF (4 mL), $POCl_3$ (2.6 mmol) was added. The mixture was stirred at room temperature for 30 min for the *in situ* formation of the acyl chloride. Then, 3,4-dimethylaniline was added to the reaction. After 1–5 h, the mixture was diluted with dichloromethane (20 mL), washed with H_2O (2 × 10 mL) and with saturated $NaHCO_3$ solution (2 × 10 mL). The organic phase was dried, filtered, and concentrated under reduced pressure. The crude product was purified by flash chromatography and crystallization. The structure was confirmed by ^1H NMR spectroscopy and mass spectroscopy.

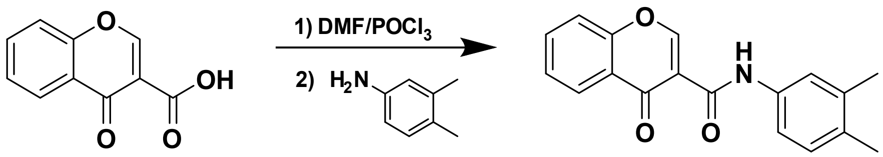

Scheme S1. Reaction between chromone-3-carboxylic acid and 3,4-dimethylaniline to obtain N-(3′,4′-dimethylphenyl)-4-oxo-4H-chromene-3-carboxamide (C27).

N-(3′,4′-Dimethylphenyl)-4-oxo-4*H*-chromene-3-carboxamide (**C27**). The compound was obtained in 23% yield and recrystallized from CH_2Cl_2: mp 233–236 °C. ^{1}H NMR ($CDCl_3$) δ = 2.25 (3H, s, CH_3), 2.28 (3H, s, CH_3), 7.12 (1H, d, J = 8.1 Hz, H5′), 7.48 (1H, dd, J = 8.1, 2.2 Hz, H6′), 7.56–7.50 (2H, m, H2′, H6), 7.59 (1H, dd, J = 8.5, 0.6 Hz, H8), 7.79 (1H, ddd, J = 8.7, 7.1, 1.7 Hz, H7), 8.34 (1H, dd, J = 8.0, 1.7 Hz, H5), 9.07 (1H, s, H2), 11.28 (1H, s, NH). ^{13}C NMR ($CDCl_3$): δ = 19.2 (CH_3), 19.9 (CH_3), 116.2 (C3), 118.0 (C8), 118.5 (C6′), 121.8 (C2′), 124.1 (C4a), 126.3 (C6), 126.5 (C5), 130.0 (C5′), 132.8 (C4′), 134.8 (C7), 135.7 (C3′), 137.2 (C1′), 156.2 (C8a), 160.5 (CONH), 162.7 (C2), 177.5 (C4). MS/EI m/z (%): 294 (M^+ + 1, 34), 293 (M^+, 88,), 173 (100), 121 (62).

Apparatus Used in NPs Analysis

The morphology of the PCL and PCL@C27 NPs were analyzed by scanning electron microscopy (SEM) (JEOL JSM-6390, Tokyo, Japan). The NPs were fixed on adequate supports and coated with carbon using a platinum sputter module (JFC-1100, JEOL Ltd.), in a high vacuum evaporator for 5 minutes at 20 mA. Observations under different magnifications were performed at 10, 15 and 20 kV.

Thermal analyses of chromone C27, PCL and PCL@C27 NPs were performed in Netzsch DSC 204 calorimeter (Netzsch, Germany). The accurately weighed sample (around 5 mg) was placed in an aluminum pan. An empty aluminum pan was used as reference. Experiments were carried out in nitrogen atmosphere (flow rate 70 mL/min) at a scanning rate of 10 °C/min in the 25-400 °C temperature range.

In Vitro Cellular Studies

Materials

Phosphate buffered saline solution (PBS 10×), nonessential amino acids (NEAA), heat inactivated bovine serum (FBS), 0.25% trypsin/1 mM EDTA, antibiotic (10,000 U/mL penincilin, 10,000 μg/mL streptomycin), Hank's balanced salt solution without and with calcium and magnesium [HBSS (-/-) and HBSS (+/+)] were purchased from Gibco Laboratories (Lenexa, KS, USA). Dulbecco's modified eagle's medium (DMEM) with 4.5 g/L glucose, hydrocortisone, ascorbic acid, 4-(2-hydroxyethyl)-1-piperazineethanesulfonic acid (HEPES), basic fibroblast growth factor (bFGF) and 3-(4,5-dimethylthiazol-2-yl)-2,5-diphenyltetrazolium bromide (MTT) were acquired from Sigma-Aldrich.

Cell Lines and Culture Conditions

Human neuroblastoma differentiated cells (SH-SY5Y cell line), epithelial colorectal adenocarcinoma cells (Caco-2 cell line) and cerebral microvascular endothelial cells (*h*CMEC/D3 cell line) were used as *in vitro* models. Neuronal SH-SY5Y cells (ATCC, Manassas, VA, USA) were routinely cultured in 75-cm^2 flasks using DMEM with 4.5 g/L glucose, supplemented with 10% heat-inactivated FBS (v/v), 1% NEAA (v/v) and 1% penicillin/streptomycin (v/v). Cells were maintained in a 5% CO_2-95% air atmosphere at 37 °C, and the medium was changed every 2 days. Cell cultures were passaged weekly by trypsinization (0.25% trypsin/1.0 mM EDTA) and used between the 19th and 28th passages, to avoid phenotypic changes. In all experiments, the cells were seeded at the density of 2.5×10^4 cells/cm^2 in cell culture medium supplemented with 10 μM retinoic acid to promote cells dopaminergic differentiation [2]. Three days after seeding, the cell culture medium was changed by new cell culture medium supplemented with 80 nM 12-*O*-tetradecanoylphorbol-13-acetate (TPA) and cells incubated for an additional 3 days period. Epithelial Caco-2 cells were obtained from the American Type Culture Collection (ATCC; Manassas, VA, USA). The cells were routinely cultured as described before, using DMEM with 4.5 g/L glucose, supplemented with 10% heat-inactivated FBS (v/v), 1% NEAA (v/v) and 1% penicillin/streptomycin (v/v), and used for all the experiments between the 42nd and 47th passages.

In all experiments, the Caco-2 cells were seeded at a density of 6 × 10^4 cells/cm^2 and used 4 days after seeding, when confluence was reached. Endothelial *h*CMEC/D3 cells were obtained from the Institut National de la Santé et de la Recherche Médicale (INSERM, Paris, France). The *h*CMEC/D3 cells (passage 35–41) were routinely grown in 75-cm^2 flasks, in EndoGRO basal medium supplemented with FBS (5%, v/v), L-glutamine (10 mM), hydrocortisone (1.0 μg/mL), ascorbic acid (50 μg/mL), heparin sulfate (1.0 μg/mL), and vascular endothelial growth factor (5 ng/mL). Cells were maintained in a 5% CO_2-95% air atmosphere at 37 °C and subcultured every 3–4 days using trypsin–EDTA (0.25% trypsin/1.0 mM EDTA) to promote detachment from the flasks. The cells were then seeded at a density of 4 × 10^4 cells/cm^2 and used 4 days after seeding, when confluence was reached.

Cellular Permeability Studies

The permeability of PCL@C6 NPs in human intestinal epithelium was evaluated using Caco-2 cells monolayer as *in vitro* model [3]. For the assay, Caco-2 cells were seeded on Transwell inserts at a cellular density of 6 × 10^4 cells/cm^2. The cell culture medium was changed every two days and the trans-endothelial electrical resistance (TEER) was measured to ensure the complete formation of the membrane using an endothelial voltammeter (World Precision Instruments, Sarasota, FL, USA). The resistance value (Ω cm^{-2}) of an empty filter was subtracted to each measurement. After the value of 800 Ω cm^{-2} was reached, PCL@C6 NPs (100 μg/mL) solution in HBSS (+/+) medium was added to the apical chamber, followed by incubation at 37 °C, in a humidified 5% CO_2–95% air atmosphere. At specific time intervals, 200 μL of basal medium was removed for C6 quantification, and the same volume of pre-heated HBSS (+/+) was added to replace the withdrawn volume. The amount of C6 presented in the basal medium was determined by measuring the fluorescence as described above. Three independent experiments were performed in triplicate and under sink conditions.

The ability of NPs to permeate the BBB was performed in *h*CMEC/D3 cells as described by Gomes et al. [4]. For that purpose, the membrane of the Transwell system (3.0 μm pore) was pre-coated with rat tail collagen type I (with a 50 μg/mL solution) for 1 hour before cells seeding, and then washed twice with PBS. Then, *h*CMEC/D3 cells were seedeed at a density of 2.5 × 10^4 cells/cm^2 on the apical side of the semi-permeable filter and maintained at 37 °C in a humidified 5% CO_2–95% air atmosphere. Cell culture medium was changed every two days and the TEER was measured with a voltohmmeter to confirm the structure of endothelial monolayer. When the TEER values reached 200 Ω cm^{-2}, PCL@C6 NPs (100 μg/mL) diluted in HBSS (+/+) were added to the apical compartment, followed by incubation, at 37 °C, in a humidified 5% CO_2–95% air atmosphere. At predetermined time intervals, 200 μL of basal medium was removed for C6 quantification, followed by its replacement by the same volume of pre-heated HBSS. The amount of C6 presented in the basal medium was determined by measuring the fluorescence as described above. Three independent experiments were performed in triplicate and under sink conditions. The data from both permeability assays was normalized with the mass of protein presented in cell monolayers, in each well. The total protein content was determined by using the Bio-Rad DC protein assay kit, according to the manufacturer's instructions. Bovine serum albumin was used as protein standard.

Statistical Analysis

Physicochemical and *in vitro* data are presented as the mean ± standard deviation (SD). GraphPad Prism V6.0 for Windows (GraphPad Sofware Inc., San Diego, CA, USA) was used to carry out the all statistical analysis. Three different tests were used to assess the normality of the data distribution: KS normality test, D'Agostino and Pearson omnibus normality test and Shapiro–Wilk normality test. Statistical significance was set at $p < 0.05$. Statistical comparisons

between groups were made using the parametric method of one-way ANOVA, followed by the Dunn's post hoc test. All assays were performed in triplicate or quadruplicate.

Results

Entrapment and Loading Efficiency

Three different nanoformulations were prepared using different amounts of C27 feeding (2.5, 5 and 10% of polymer mass). After ultrafiltration process, which guarantee the remove of C27 non-encapsulated, the nanoformulations were ultracentrifuged and the resulting powder was analysed for the C27 concentration (C_{C27}) by UV-Vis. The results calculated for encapsulation efficiency (EE%) and drug loading capacity (DLC%) were presented in Table S1.

Table S1. Values of EE% and DLC% obtained for different formulations prepared with different amounts of feeding C27.

Nanoformulation	C27/Polymer (w/w, %)	EE%	DLC%
PCL NPs	0	---	---
PCL@C27 NPs	2.5	46.1 ± 2.1	1.13 ± 0.12
	5	62.5 ± 2.3	2.96 ± 0.11
	10	12.7 ± 2.3	1.16 ± 0.22

All measurements were performed in triplicated and the results are presented as mean ± SD.

From all the nanoformulations prepared, it was possible verified that highest EE% (62.5 ± 2.3%) and DLC% (2.96 ± 0.11%) was achieved when 5% (w/w, weight of polymer) of C27 was used in the preparation of PCL@C27 NPs.

Morphologic Characterization of PEGylated PCL NPs

The D_{DLS}, polydispersity index (PdI) and zeta potential (ZP) of empty PCL NPs, PCL@C27 NPs and PCL@C6 NPs were assessed in Milli-Q water and PBS (1×). The results are presented as mean ± SD in Table S2.

Table S2. Comparison of NPs size and z-potential values in Milli-Q, PBS (1×, +/+) and HBSS media (+/+).

Formulation	Medium	D_{DLS} (nm)[a]	PdI[b]	Z-potential (mV)[c]
PCL NPs	*Milli-Q*	197.2 ± 10.7	0.08 ± 0.02	-14.8 ± 0.9
	PBS (1×, +/+)	193.0 ± 7.8	0.10 ± 0.05	- 8.2 ± 1.1
	HBSS (+/+)	195.7 ± 12.0	0.13 ± 0.02	- 6.5 ± 0.5
PCL@C27 NPs	*Milli-Q*	213.3 ± 14.9	0.11 ± 0.01	-14.0 ± 0.5
	PBS (1×, +/+)	210.8 ± 16.0	0.12 ± 0.04	- 6.4 ± 0.7
	HBSS (+/+)	213.5 ± 17.6	0.14 ± 0.06	- 5.3 ± 0.2
PCL@C6 NPs	*Milli-Q*	200.1 ± 10.6	0.04 ± 0.01	-15.3 ± 0.7
	PBS (1×, +/+)	196.9 ± 7.7	0.16 ± 0.03	- 6.5 ± 0.2
	HBSS (+/+)	200.6 ± 16.1	0.16 ± 0.04	- 5.3 ± 0.2

[a,b,c] Values determined by dynamic light scattering (DLS) in Milli-Q water, PBS (1×, +/+) and HBSS media (+/+). [b] Polydispersity Index. All measurements were performed in triplicated and the results are presented as mean ± SD.

The dynamic light scattering (DLS) analysis allowed to realise that all nanoformulations presented similar low PdI (<0.16) and monodisperse profiles with hydrodynamic sizes (D_{DLS}) between 193 and 213 nm. A non-significant difference was found in both analysis. However, zeta potential (ZP) values are affected by the medium where NPs are dispersed, since for the ion containing medium (PBS or HBSS), all nanoformulations demonstrated ZP values lower in absolute value when compared with data obtained in Milli-Q water. Overall, all nanoformulations showed size and stability in physiological conditions suitable for further studies in *in vitro* and *in vivo* models.

UV-Vis Spectroscopy of Chromone C27

UV-Vis spectra of C27 was acquired with a Shimadzu UV-Vis spectrophotometer (UV-1700 PharmaSpec, Japan) (Figure S1).

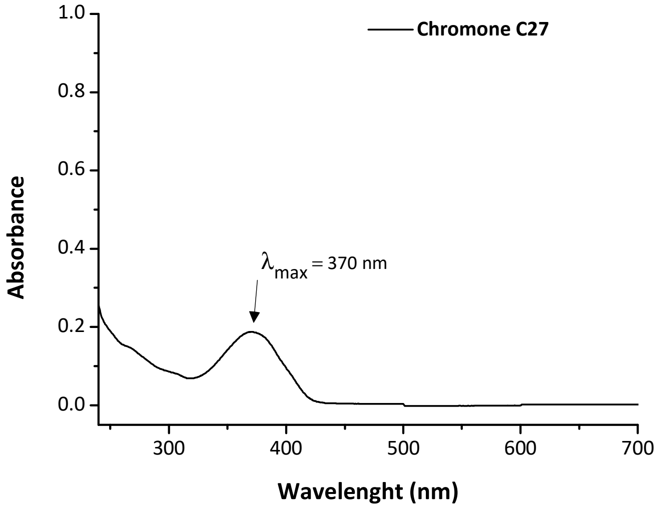

Figure S1. UV-Vis spectra of chromone C27 (50 μM) in DMSO.

Chromone C27 was dissolved in DMSO at final concentration of 50 μM and the UV-Vis spectra was obtained between 200 and 700 nm. The data from UV-Vis analysis showed a maximum absorbance at 370 nm.

References

1. Reis, J.; Cagide, F.; Chavarria, D.; Silva, T.; Fernandes, C.; Gaspar, A.; Uriarte, E.; Remião, F.; Alcaro, S.; Ortuso, F.; et al. Discovery of New Chemical Entities for Old Targets: Insights on the Lead Optimization of Chromone-Based Monoamine Oxidase B (MAO-B) Inhibitors. *J. Med. Chem.* **2016**, *59*, 5879–5893, doi:10.1021/acs.jmedchem.6b00527.
2. Fernandes, C.; Pinto, M.; Martins, C.; Gomes, M.J.; Sarmento, B.; Oliveira, P.J.; Remião, F.; Borges, F. Development of a PEGylated-Based Platform for Efficient Delivery of Dietary Antioxidants Across the Blood–Brain Barrier. *Bioconj. Chem.* **2018**, *29*, 1677–1689, doi:10.1021/acs.bioconjchem.8b00151.
3. van Breemen, R.B.; Li, Y. Caco-2 cell permeability assays to measure drug absorption. *Expert Opin. Drug Metab. Toxicol.* **2005**, *1*, 175–185, doi:10.1517/17425255.1.2.175.
4. Gomes, M.J.; Fernandes, C.; Martins, S.; Borges, F.; Sarmento, B. Tailoring Lipid and Polymeric Nanoparticles as siRNA Carriers towards the Blood-Brain Barrier—From Targeting to Safe Administration. *J. Neuroimmune Pharmacol.* **2017**, *12*, 107–119, doi:10.1007/s11481-016-9685-6.
